# Supplementary material for: Deciphering ovarian cancer heterogeneity through spatial transcriptomics, single-cell profiling, and copy number variations
Source: PLoS One. 2025 Mar 4;20(3):e0317115. doi: 10.1371/journal.pone.0317115 (PMC11878925; doi:10.1371/journal.pone.0317115)
Supplement: S1 Fig — (PDF) [file pone.0317115.s001.pdf]

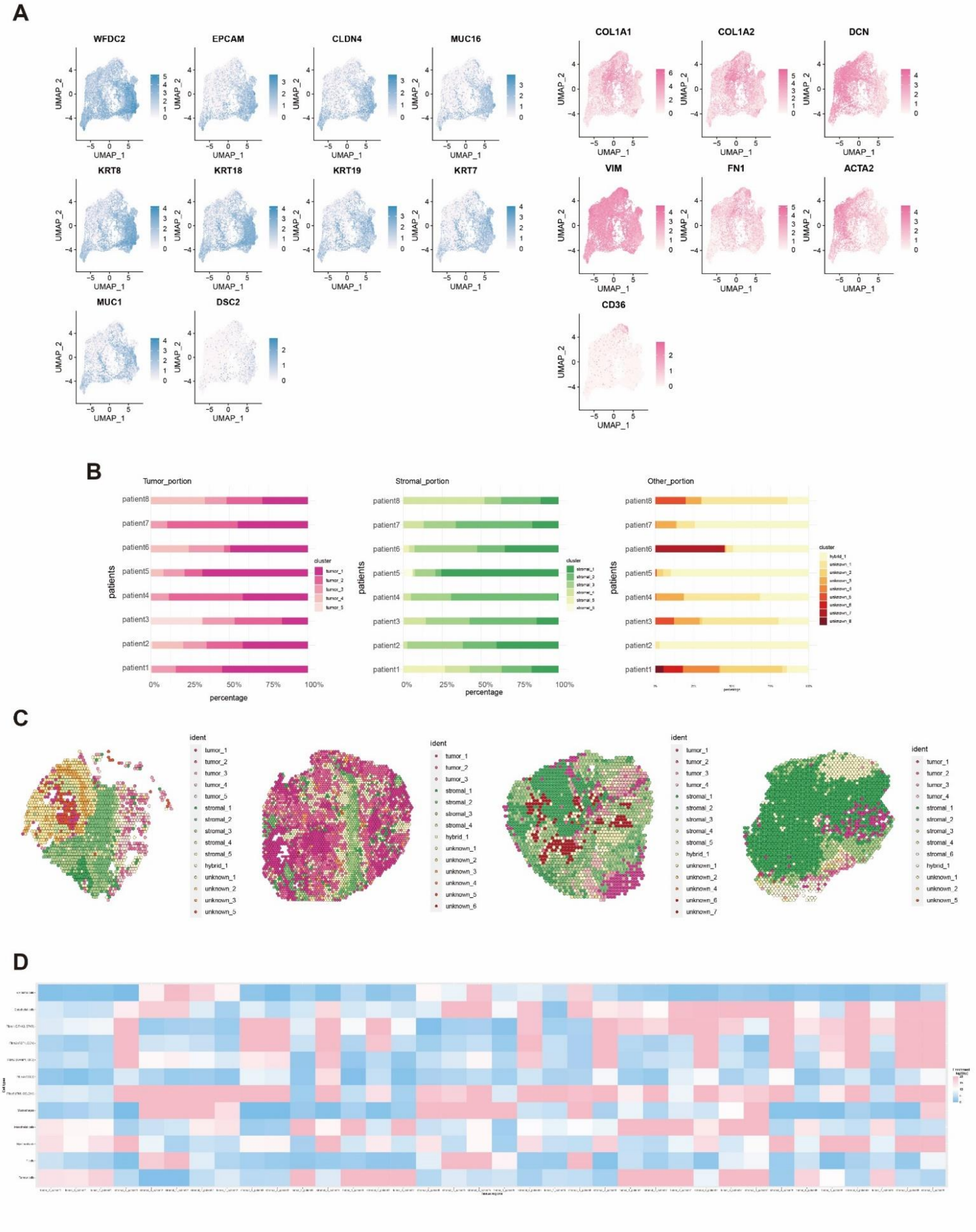

Figure S1

A: UMAP plot of the expression of tumor (blue) and stromal (pink) markers. B: tumor (left), stromal (middle), and unknown (right) cluster distributions in each patient. C: Spatial distribution of clusters in patients (from left to right: patient 3, patient 7, patient 6, patient 5). E: heatmap of cell enrichment in the clusters of ST data.

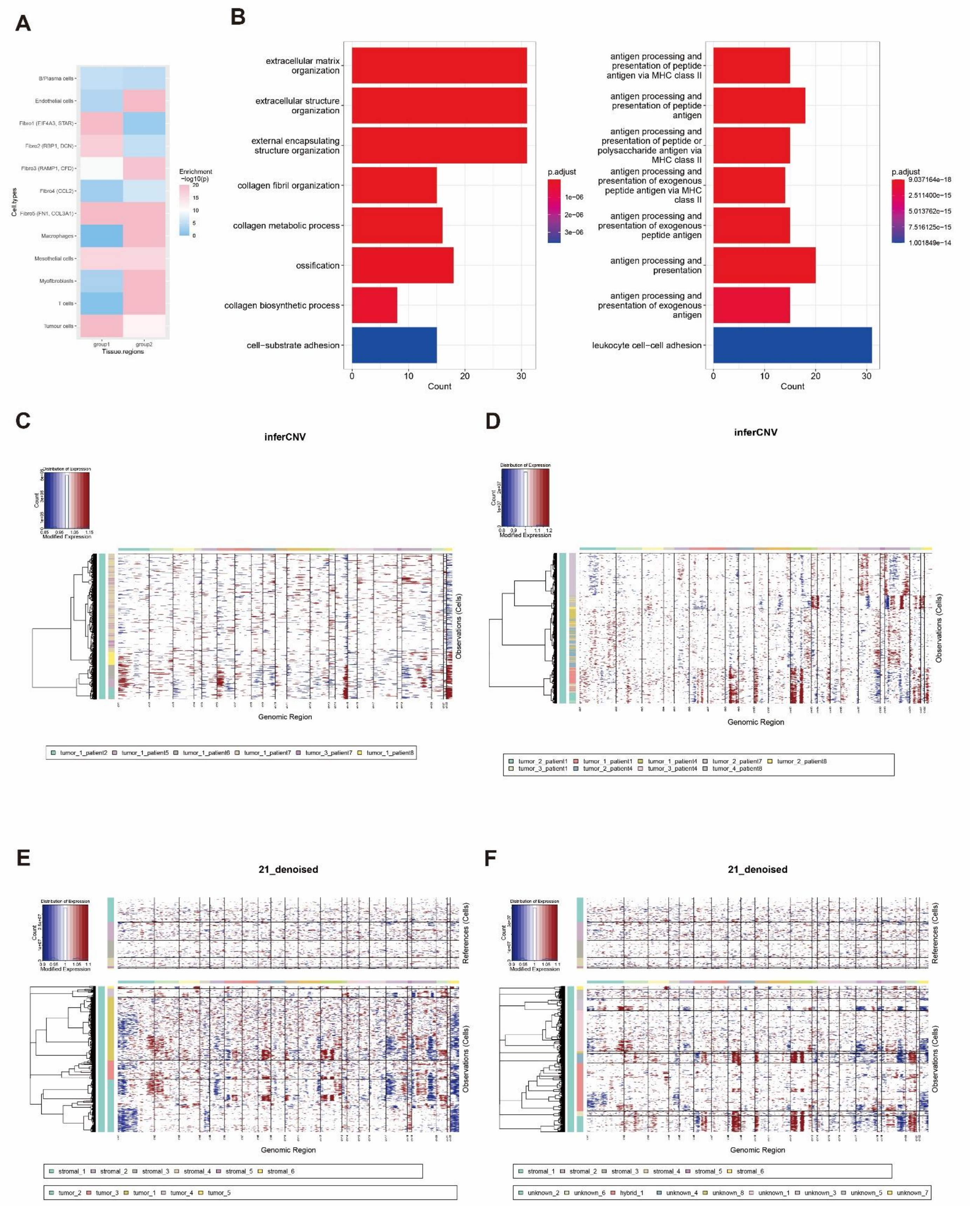

Figure S2

A: cell enrichment of group 1 and group 2 stromal clusters. B: pathway activity of group 1 (left) and group 2 (right) of stromal clusters. C, D: heatmap of CNVs in group 1(C) and group 2(D) of tumor clusters. E, F: heatmap of CNVs in tumor (E) and unknown clusters (F)

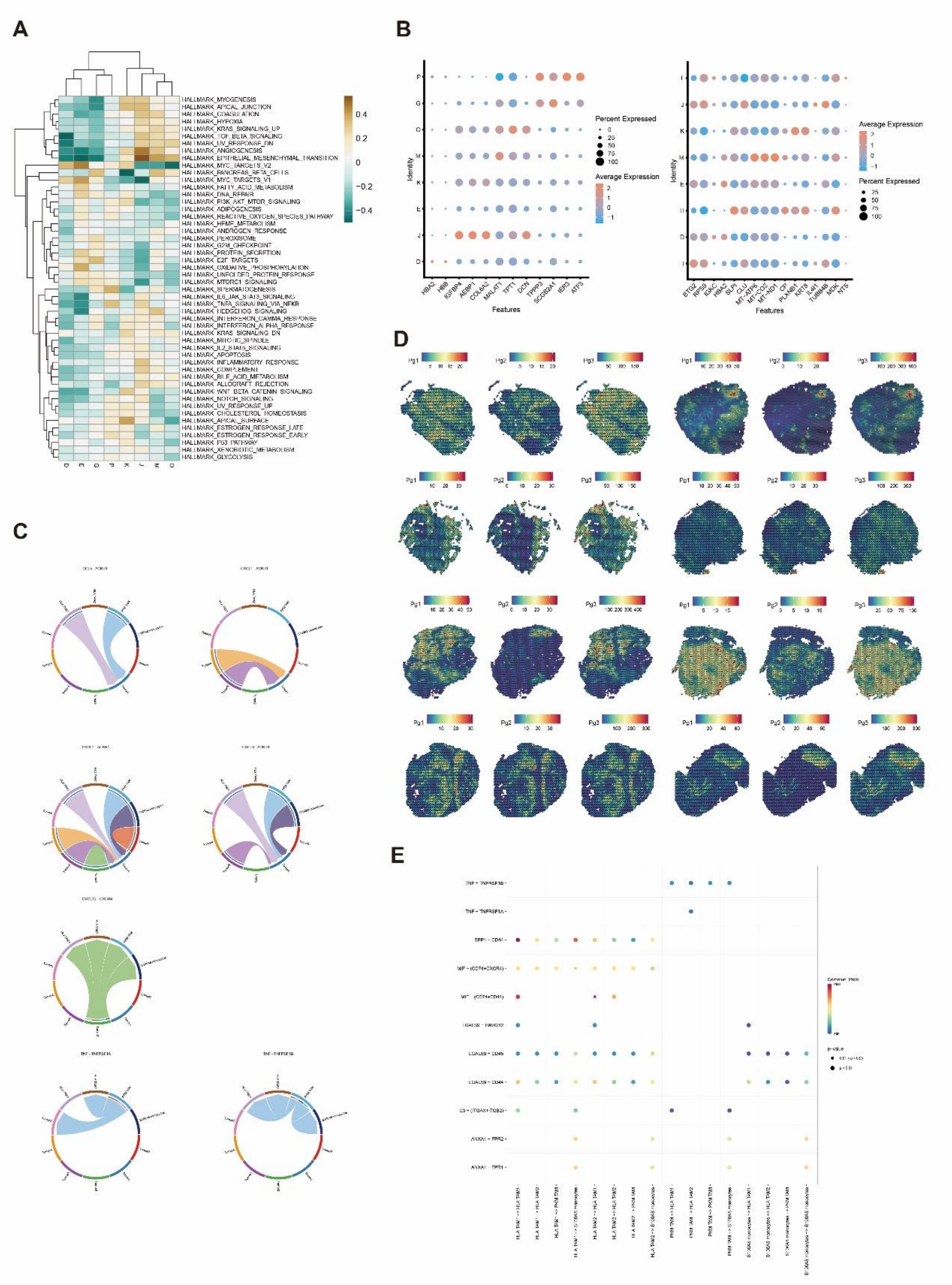

Figure S3

A: heatmap of pathway activity of different clones in patient 4. B: dot plot of top differential expressed genes in patient 4 (left) and patient 8 (right). C: chord plot of important ligand-receptor pair in the communication. D: spatial visualization of the enrichment of metaprograms. E: dot plot of cell-cell communication between macrophages.

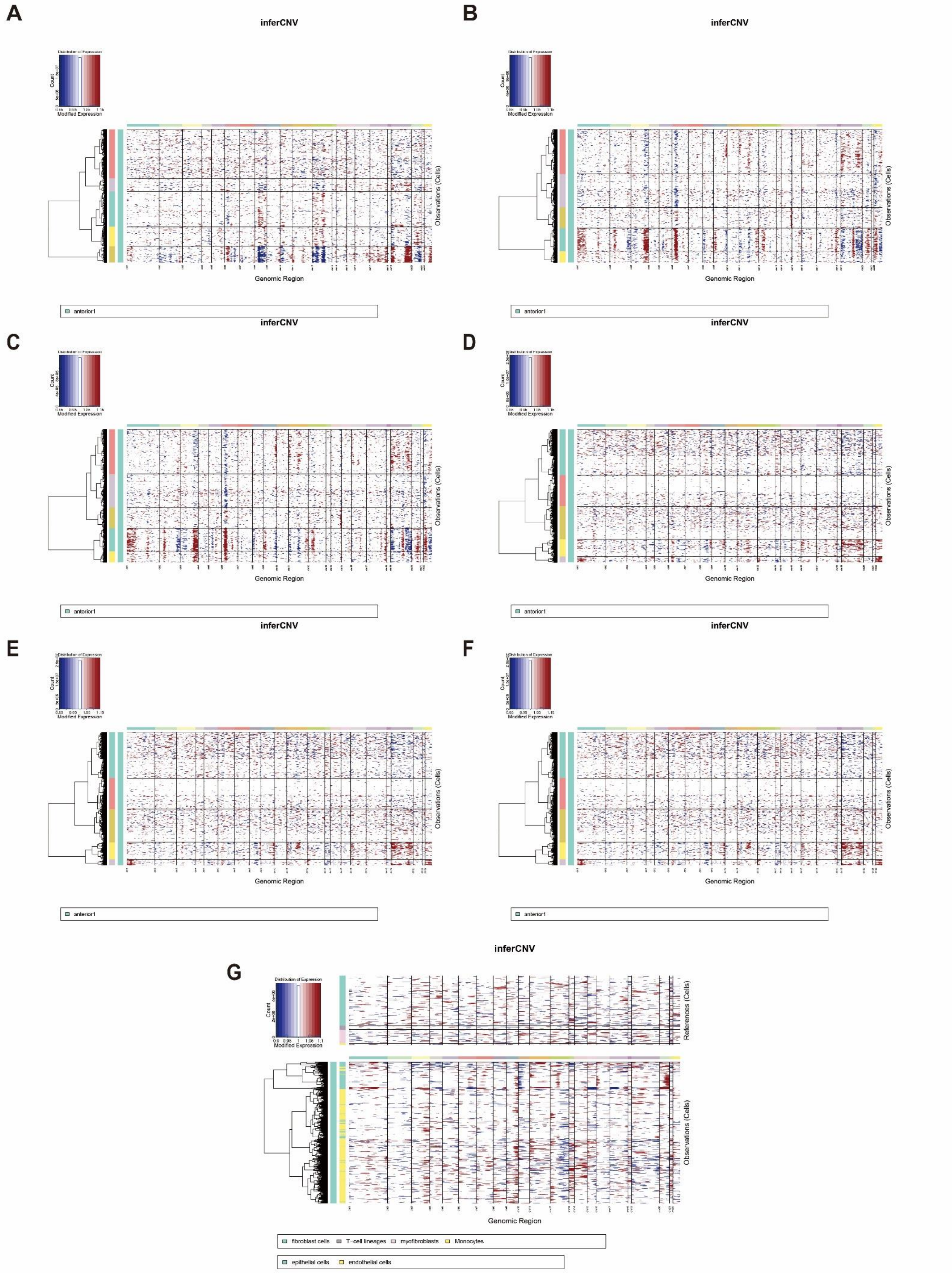

Figure S4

A-F: heatmap of CNVs in ST data in different patients (A: patient 1, B: patient 2, C: patient 3, D: patient 5, E: patient 6, F: patient 7). G: heatmap of CNVs in scRNA seq data

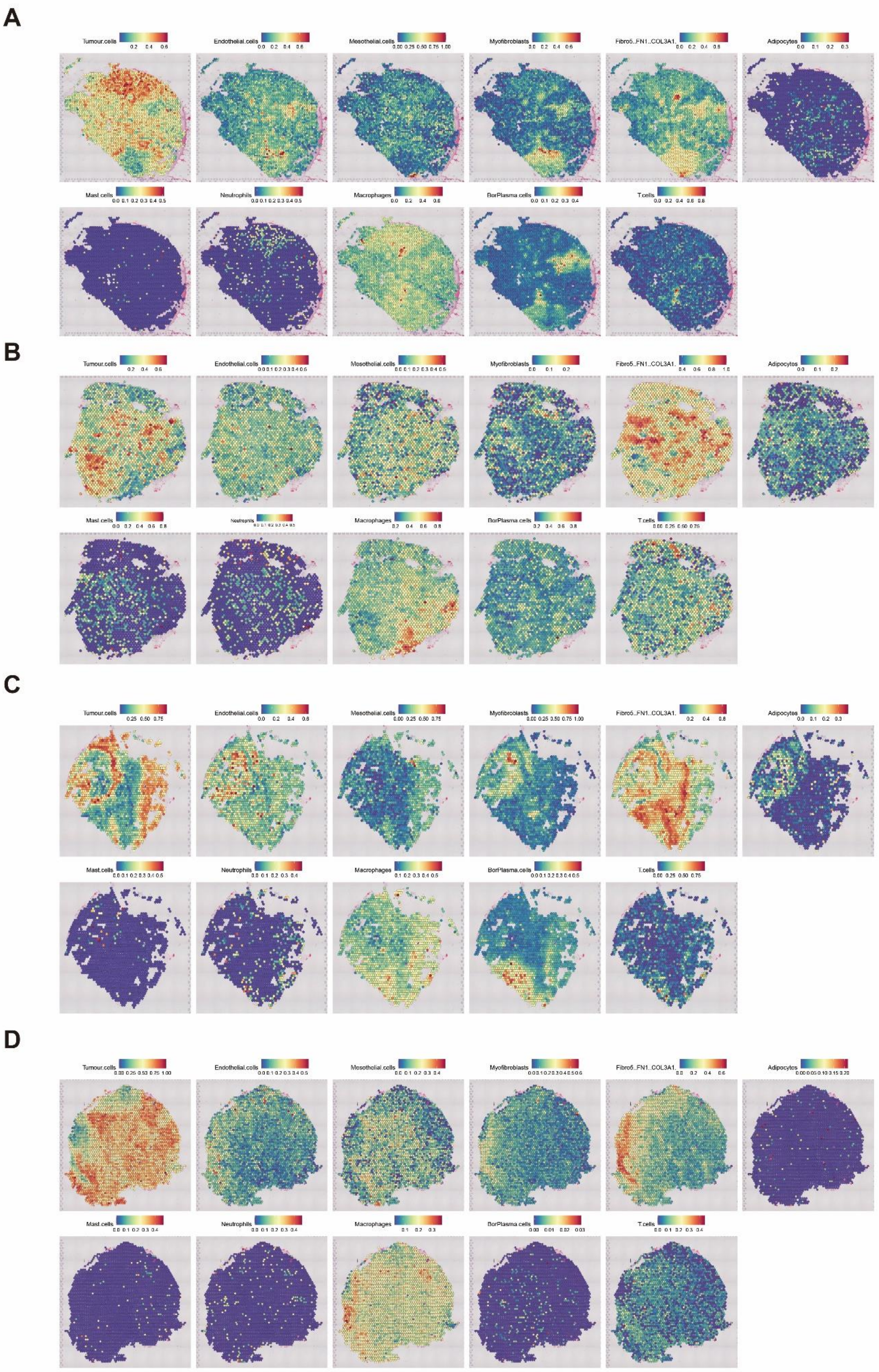

Figure S5

A-D the normalized enrichment of different cell types from patient 1 to patient 4

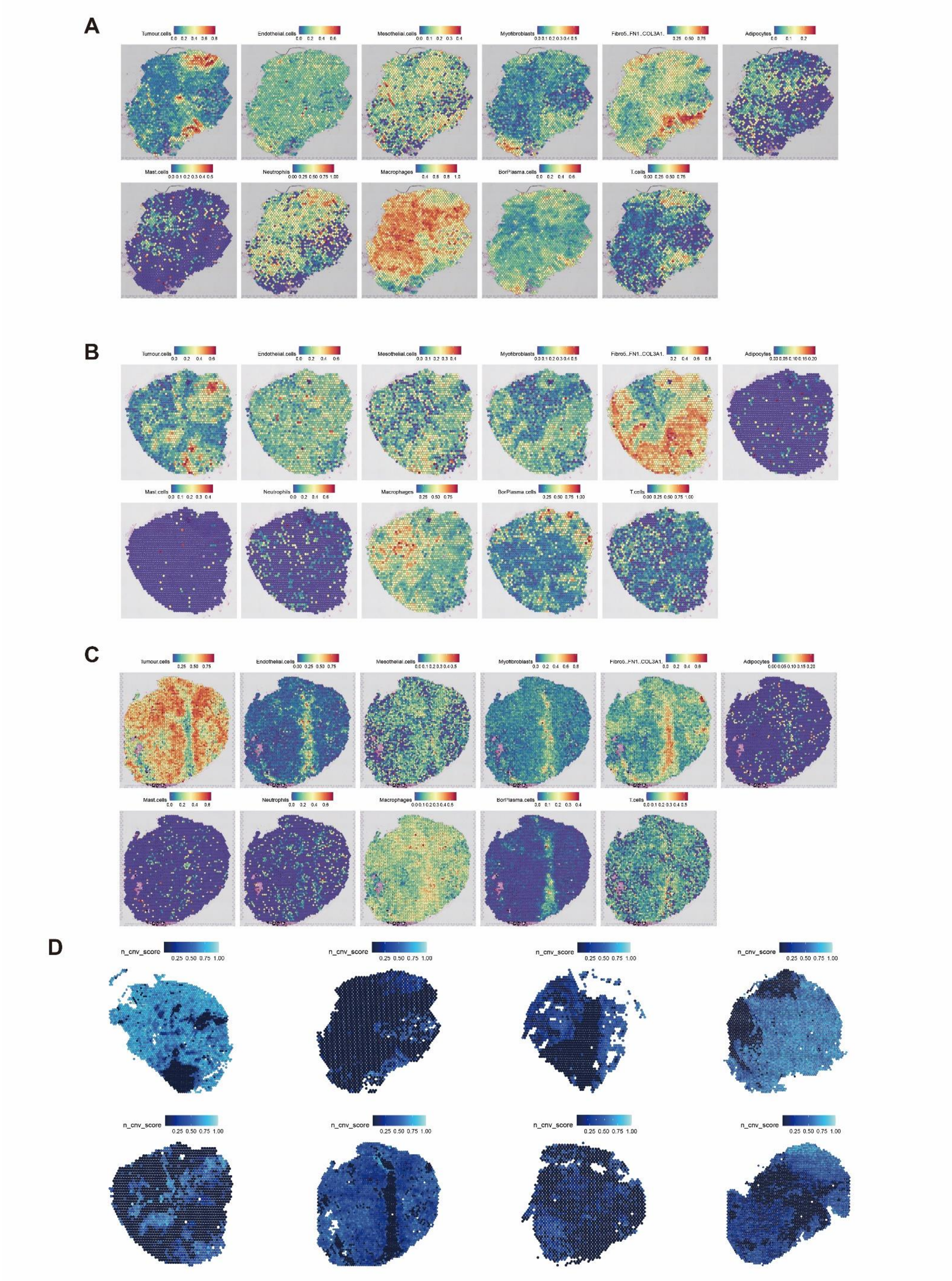

Figure S6

A-C: the normalized enrichment of different cell types from patient 5 to patient 7. D: normalized CNV scores in patients 1-7

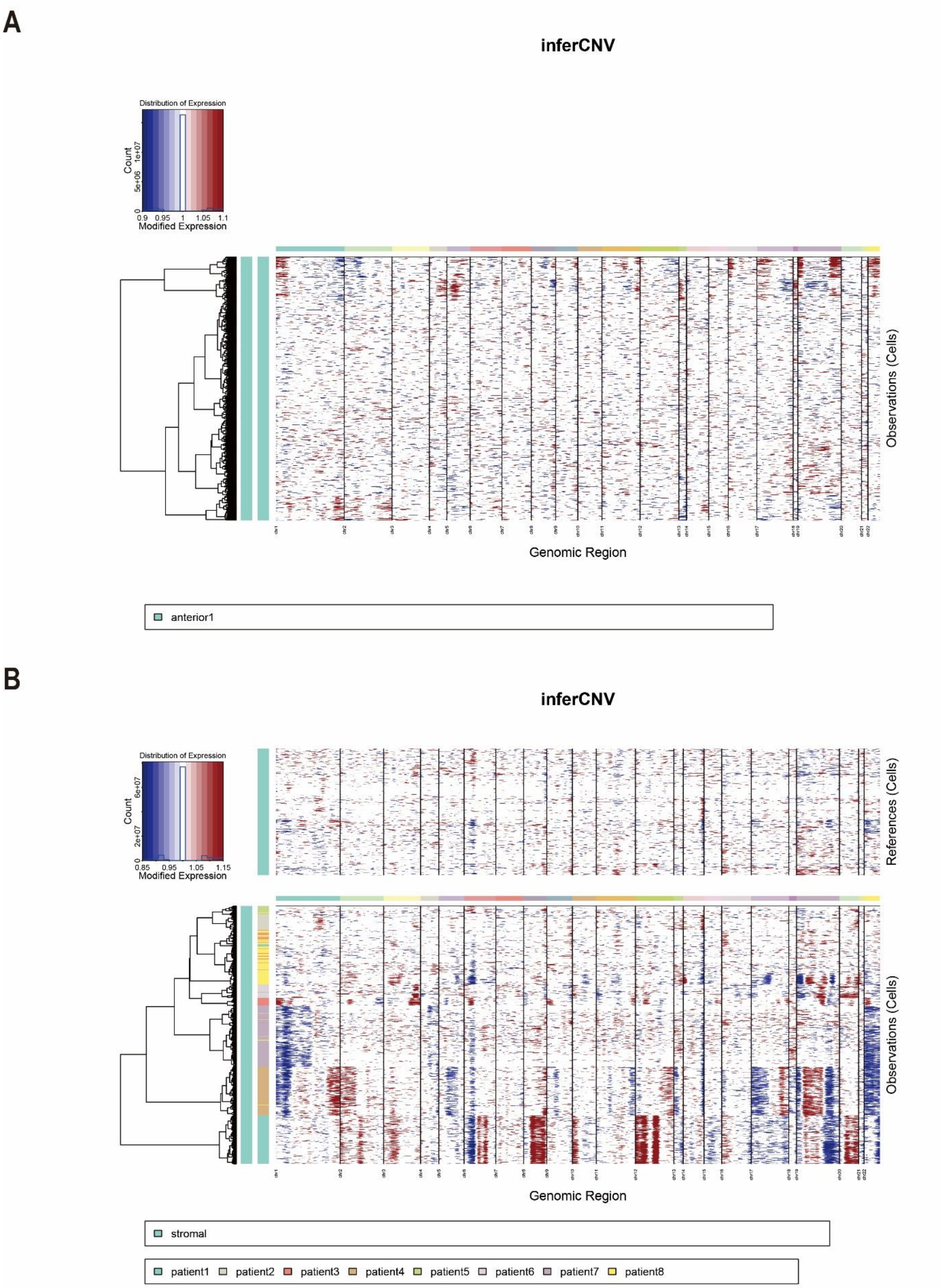

Figure S7

A): Heatmap of copy number variations (CNVs) in patient 4. B: Heatmap of CNVs across all the patients

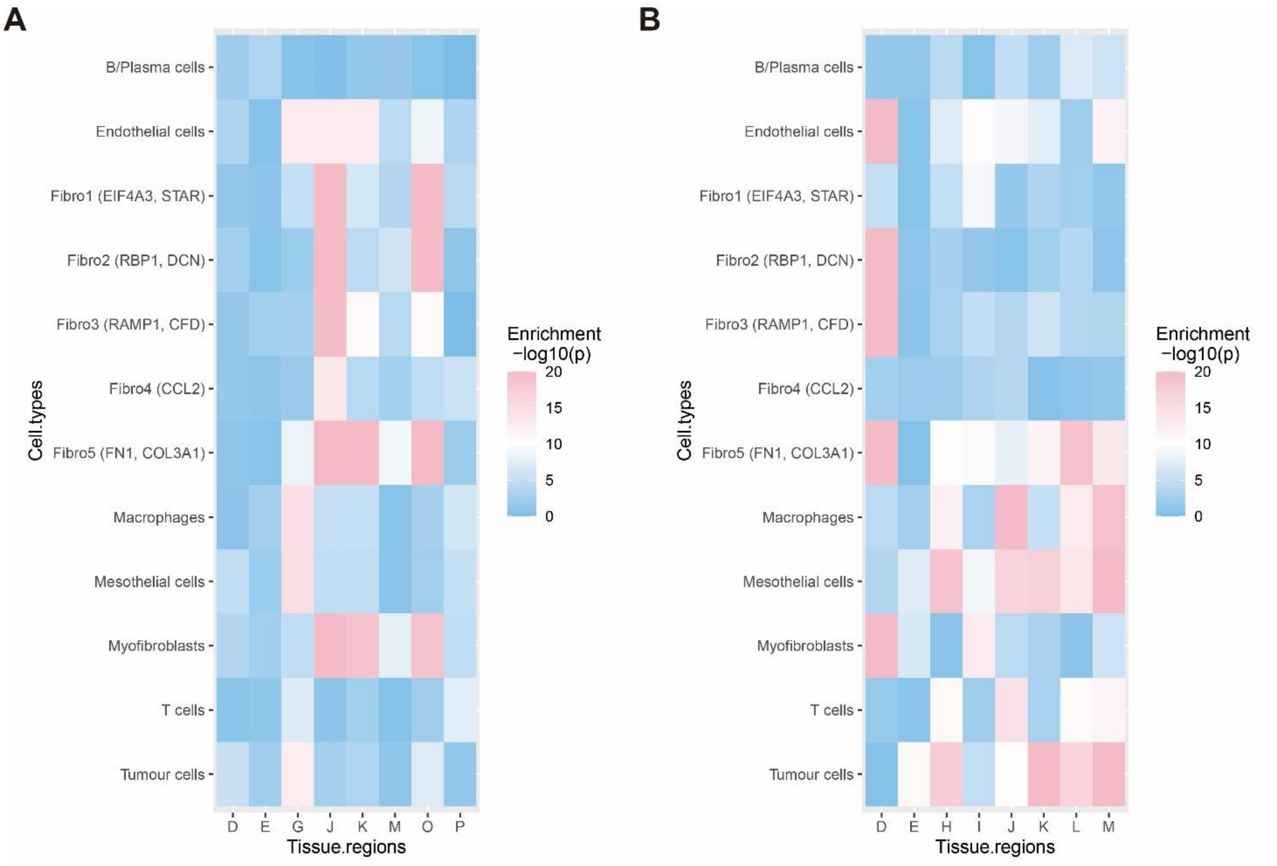

Figure S8

A: Immune infiltration of different clones in patient 4. B: immune infiltration of different clones in patient 8

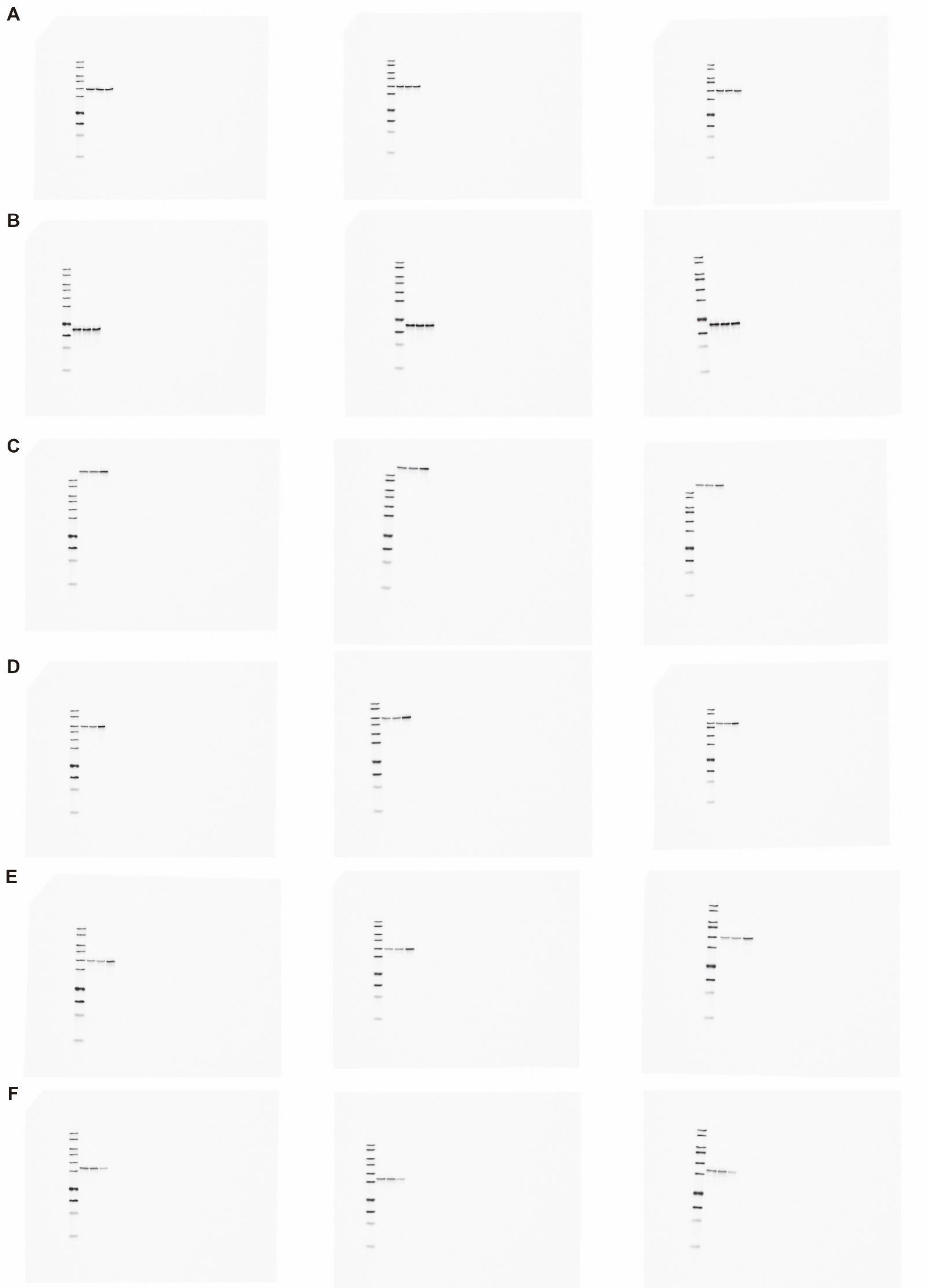

Figure S9:

the original image of western blot(N=3) of AKT(A), GADPH(B), Ki67(C), NCL(D), pAKT(s473) (E) and PTEN(F)
